# The physical activity implications of retirement across occupational activity groups

**DOI:** 10.1016/j.ypmed.2023.107570

**Published:** 2023-06-13

**Authors:** Leonie Glasson, Jenna Panter, David Ogilvie, Richard Patterson

**Affiliations:** 1 MRC Epidemiology Unit, University of Cambridge School of Clinical Medicine, Cambridge, UK

## Abstract

Retirement is an important later life transition which may represent a critical period for physical activity in older age. Past findings on the association between retirement and physical activity are inconclusive and there is some evidence that the physical activity implications of retirement may differ by occupational activity level. This study used data from waves 4–9 (June 2008–July 2019) of the English Longitudinal Study on Aging to evaluate whether there is an association between retirement and physical activity, and whether this varies across occupational activity groups.

Retirement was associated with a significant increase in physical activity (n=10 693; β: 0.602 METhrs/wk [95% CI: 0.490, 0.713], p<0.001). There were significant interactions between retirement and past occupational activity level (n= 5 109; X_2_(3)=32.59, p<0.001), such that people retiring from sedentary or standing occupations experienced a significant increase in physical activity with retirement but retirement from an occupation involving heavy manual labour was associated with a decrease in physical activity.

This study quantified the importance of retirement for later life physical activity. With demographic aging, the population health importance of later life physical activity will likely become more important. These findings should inform the design of public health interventions to increase physical activity around the retirement transition.

## Introduction

The health benefits of physical activity are broad and well documented and may be especially powerful in later life when the burden of ill-health is often most severe (1–4). Despite these benefits, inactivity remains a global problem. A Lancet review commissioned by the WHO in 2017 estimated that 28% of the global population did not meet global physical activity recommendations of at least 150 minutes of moderate-intensity or 75 minutes of vigorous-intensity physical activity per week (5). Physical inactivity is particularly high among older adults; in England an estimated 56% of people aged over 65 failed to meet physical activity recommendations in 2016 (6). Reducing the prevalence of physical inactivity is a global priority. In 2018 the WHO launched a Global Action Plan on Physical Activity, which recommended that physical activity is increased across all societal sectors (1). In the UK, increasing physical activity is recognized as an explicit target in the governmental consultation document on preventative health in the 2020s and forms a critical part of the UK Tackling Obesity Strategy (7,8).

Retirement is an important milestone in the life-course and may represent a critical transition period for physical activity engagement in association with changes in socialisation, income, mobility and time-use (9). Past reviews on the association between retirement and physical activity have generally reported an overall increase in leisure-time PA, walking, and domestic activities, but a decline in occupational activity and active forms of transport over the retirement transition (10–15). However, changes in total physical activity are inconsistent between reviews and appear to be moderated by a number of sociodemographic and occupational factors.

Past occupational activity level has been found to be an important moderating factor in the association between retirement and physical activity. In a systematic review, published in 2012, Barnett *et al*. reported that retiring from manual or “low-grade” occupations was associated with a decline in physical activity over the retirement transition, however this association was reversed for people retiring from sedentary or high-grade occupations (13). These findings have since been replicated in subsequent reviews (10,11,15). In general, work is continuing to shift towards more sedentary, office-based occupations, thus making the need for up-to-date data on occupational activity especially important.

This study used detailed survey data collected biennially from an ongoing cohort of older adults in England. This minimised the period between data collection and retirement, therefore reducing the impact of confounding factors or events. The richness of the data enables the inclusion of putative confounders for which data is not often available, thus helping minimize the distortive effect that uncontrolled confounding may have on reported associations. The large sample of older adults enabled us to assess whether there is an association between retirement and PA, and to explore whether this association differs by several individual-level characteristics, including sex and past occupational activity level.

## Methodology

This study used data from waves 4–9 inclusive (June 2008–July 2019) of the English Longitudinal Study of Aging (ELSA) (16). The ELSA cohort was established in 2002 and consisted of over 18,000 English residents aged 50 years or older. Participants were contacted biennially for data collection, through computer-assisted personal interviews, self-completion questionnaires, and life-history interviews. Ethical approval was gained separately for each wave of ELSA, e.g., Wave 9 received ethical approval from the South Central – Berkshire Research Ethics Committee (17/SC/0588). All participants provided informed consent and data collection was performed in accordance with the Declaration of Helsinki.

The primary exposure variable was self-reported retirement status. Responses were dichotomized into *‘fully retired’* or *‘employed’*. Participants responding as *‘semi-retired’* were classified as *‘employed’*, and those reporting themselves as *‘unemployed’, ‘permanently sick or disabled’, ‘looking after home or family’* or *‘other’* were not included for that wave. Participants were classified as having retired over the study period if they transitioned from being *‘employed’* to *‘fully retired’* and any intermediate waves were disregarded.

The primary outcome was self-reported physical activity assessed via three questions about the frequency and intensity of activity undertaken by each participant. Participants were shown examples of mild, moderate, and vigorous activities (Table S1) and were asked how frequently they undertook each intensity of activity from options of *‘more than once a week’, ‘once a week’, ‘one to three times a month’*, or *‘hardly ever, or never’*. The ELSA physical activity questionnaire has been validated using objective accelerometer-based measures of physical activity and has been associated with a range of outcomes, including healthy aging, cardiovascular disease and mortality (17,18).

A continuous measure was calculated by applying a metabolic equivalent (MET) score to each intensity of activity and multiplying by the reported frequency of activity per week (Equation S1). A MET score is the ratio of estimated metabolic rate associated with an activity to the resting metabolic rate. One MET is approximately equivalent to the energetic cost of sitting quietly. Drawing on MET ranges stated in The Compendium of Physical Activities, a MET of 9.0 was applied to vigorous activity, 4.5 to moderate activity and 2.0 to mild activity (19). This is also consistent with WHO physical activity guidelines, which equate 150 minutes of vigorous activity to 300 minutes of moderate activity, i.e. a two to one conversion rate (1). To calculate METhrs/week, each activity was assumed to be 0.5 hours long (Equation S1).

To ensure that the assumptions used to calculate the outcome did not substantially impact the finding, we generated a second, categorical measure of physical activity. This measure combined the frequency and intensity of activity to generate an overall physical activity level with four categories: *high, moderate, low*, and *inactive* (Table S3). A similar categorization has been used previously with this dataset, demonstrating a robust dose-response association with mortality (20–23). The continuous and categorical classifications of physical activity were highly correlated (r = 0.878) indicating high correspondence between these indices.

To test whether the association between retirement and physical activity varies by occupational activity level, the self-reported activity level of a participants last occupation prior to retirement was determined. This variable took values of *‘sedentary’, ‘standing’, ‘physical work’*, or *‘heavy manual labour’*, and was derived from an ELSA question that asked participants to choose the level of physical activity of their main job from the four categories listed above.

Time-varying covariates were participant age (years), marital status (married or civil partnership/not married), total net (non-pension) wealth quintile, current smoking status (yes/no), alcohol consumption in the last 12 months (some/none), self-reported health (poor/fair/good/very good/excellent), and mobility (difficulty walking 100 yards: yes/no). Age and self-reported health were assumed to have a linear trend effect, which were checked using margins plots. This study focused on intra-individual change in physical activity and therefore time-invariant factors, such as sex, ethnicity and education were not included.

### Statistical Analyses

The characteristics of participants who retired over the study period were compared to those of participants who remained employed and those who were retired throughout. To descriptively compare participants over the retirement transition, the characteristics of participants retiring were compared on their last wave before retirement and first wave after retirement. Differences were assessed with two sample t-tests, ANOVA or Pearson’s chi-squared tests, as appropriate.

We used fixed effect regression models to investigate changes in physical activity over retirement. When physical activity was coded as a categorical variable (inactive/low/moderate/high) a fixed-effect multinomial logistic regression model was fitted to quantify the association between retirement and physical activity. When physical activity was coded as a continuous variable in METhrs/wk, a fixed effects linear regression model was fitted. Multicollinearity between covariates was tested through a correlation matrix. Missingness was generally low across all variables, so regression models were fitted by complete case analysis.

To explore differences in the association between retirement and physical activity by sex and occupational activity, interaction terms were fitted. Occupational activity was assessed using the last level of occupational activity prior to retirement. Likelihood ratio tests were used to compare models with and without the interaction term. Where there was evidence for effect modification, analyses were stratified by the effect modifier.

Sensitivity analyses were conducted to evaluate the effect of the month of questionnaire completion and the wave of analysis, on the association between retirement and physical activity. Sensitivity analyses were also conducted to assess the effect of reversible retirement (participants who retired and then returned back to employment) and on the classification of patients who self-identified as “semi-retired”.

All analyses were conducted in STATA SE 17. Confidence intervals were set at the 95% level with 2-sided P-values used throughout.

## Results

### Baseline Descriptive

**Figure 1:**
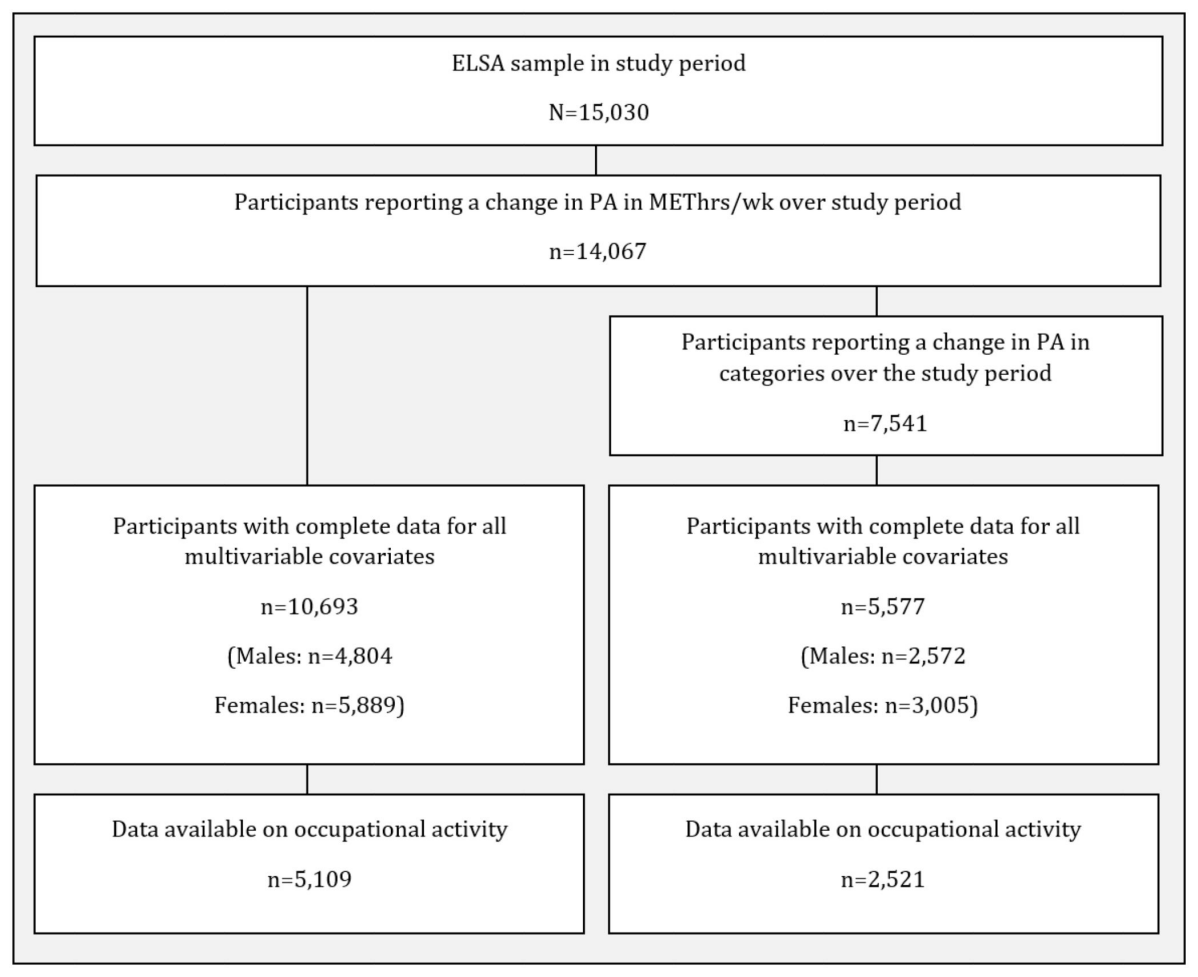
Flowchart of sample size for analyses.

A total of 14,067 participants had at least two waves of data on reported a change in physical activity in METhrs/wk over the study period, of which 10,693 (4,804 males, 5,889 females) had complete data for all the time-varying covariates. 7,541 participants reported a change in physical activity which lead them to be classified in another physical activity category. Of these, 5,577 (2,572 males, 3,005 females) participants had complete data for all covariates (Figure 1).

The percentage and patterns of missingness for each prior listed covariate were evaluated (Table S4). The greatest missingness was for wealth and alcohol consumption (both 15%). Observations with missing data differed significantly from observations with complete data in age, ethnicity, marital status and mobility, however the magnitude of difference was generally small (Table S5).

The average age of participants across the total sample was 64.7 years. Of 10,693 participants in the study sample, just over a fifth (21%) retired over the study period; the rest were either retired throughout (54%) or remained employed (25%) (Table 1). Participants who retired in the study period were on average 11 years younger than participants who were retired throughout the study period, were more likely to be in the highest wealth quintile (27% vs 21%), report excellent health (18% vs 9%) and less likely to have mobility difficulties (3% vs 15%). Compared to those remaining employed, participants retiring over the period were on average 5.1 years older, were more likely to be in the highest wealth quintile (27% vs 19%), and were more likely to be married or be in a civil partnership (74% vs 67%). participants who were employed throughout reported higher levels of physical activity (6.99 vs 6.78 METhrs/wk) than those who were retired throughout- this is likely in reflection of the younger average age for employed participants (55.0 vs 60.1 years).

Of the 2,199 participants retiring over the study period, 2,001 had complete data for at least one wave before retirement and one wave after retirement. After retiring, participants were on average older (64.7 vs 63.1; p<0.001) and more likely to have mobility difficulties (5% vs 3%; p=0.014) (Table 2). Physical activity (METhrs/wk) was significantly higher in the first wave after retirement compared to the last wave before retirement (7.0 vs 6.7 METhrs/wk; p=0.022).

### The association between retirement and PA

There was no evidence to suggest that the association between retirement and physical activity differed significantly by sex (X_2_(1)=0.710, p=0.399) so subsequent analyses were not stratified by sex.

In the maximally adjusted model, there was evidence that retirement was associated with a significant increase in METhrs per week of physical activity completed (n=10 693; β: 0.602 [95% CI: 0.490, 0.713], p<0.001) (Model 5 - Table 3).

Age was an important confounder in the association between retirement and physical activity. As expected, increasing age was associated with a significant increase in the likelihood of retirement (β [95% CI], 0.657 [0.545, 0.770]) and a decrease in physical activity independent of retirement (β [95% CI], -0.122 [-0.131, -0.113]) (Table 3). Further adjustment for wealth, health and mobility and behavioural characteristics led to a slight attenuation in the association between physical activity and retirement (β [95% CI], 0.602 [0.490, 0.713]). The association between retirement and physical activity was consistent for the continuous and categorical measures of physical activity (Table S7).

### The effect of past occupational activity level

Data on the occupational activity level of a participant’s last job were only available for participants who were employed for at least one wave. For the linear regression model, 5,109 participants had available data on occupational activity. When physical activity was coded as a categorical variable, 2,521 participants had available data on occupational activity.

Most participants had a sedentary last occupation (36%) and only 7% of participants reported that their last occupation involved heavy manual labour (Table 4). Compared to the total sample, participants retiring from heavy manual labour were more likely to be male (85% vs 49%), be a current smoker (23% vs 15%), be in the lowest wealth quintile (19% vs 13%) and have no educational qualifications (25% vs 14%).

There was a significant interaction between retirement and past occupational activity level (X_2_(3)=32.59, p<0.001). After adjusting for putative confounders, retiring from a sedentary or standing occupation was associated with an increase in physical activity (β: 0.580 [95% CI: 0.375, 0.785], p<0.001; β: 0.330 [95% CI: 0.095, 0.565], p = 0.006) (Table 5). However, retiring from an occupation involving heavy manual labour was associated with a decrease in physical activity levels (β: -0.823 [95% CI: -1.440, -0.206], p = 0.009). Consistent results were obtained from fixed-effect multinomial regression with physical activity coded as a categorical variable (Table S8). These findings are consistent with changes in the proportion of participants meeting UK physical activity recommendations (11 METhrs/week): the proportion of sedentary workers meeting these recommendations rose from 46% before retirement to 54% after, and a comparable decrease (from 57% to 43%) was observed for those retiring from heavy manual labour (Table S9).

### Sensitivity Analyses

Neither the month of questionnaire completion nor wave number significantly affected the magnitude or direction of the association between retirement and physical activity (Tables S10, S11).

277 participants retired and then returned to employment, however these participants did not differ (for the majority of sociodemographic variables considered) from participants who remained retired (Table S12). Excluding these participants did not substantially affect the findings (Table S13).

Semi-retired participants fell between employed and retired people for the majority of the sociodemographic variables considered, but were generally more similar to employed individuals than retired individuals (Table S14). Analyses excluding semi-retired participants were consistent with our main analyses. (Table S15).

## Discussion

### Principal findings

After adjusting for putative confounders, retirement was associated with an increase of 0.6 METhrs/wk. The association between retirement and physical activity differed by past occupational activity level. Retirement from a sedentary or standing occupation was associated with a significant increase in physical activity however retirement from a manual occupation was associated with a significant decrease in activity. We found no differences between men and women. This is an interesting finding given consistent findings of higher physical inactivity in females than males (5) and gender differences in occupation types (24). The increase in physical activity reported in this study is consistent with previous findings for leisure time PA, however literature on the effect of retirement on total physical activity has previously been inconsistent (10,11,14,15,25). Under the assumptions made in this study, an increase of 0.6 METhrs/wk corresponds to approximately 18 minutes more mild activity (2 METS, e.g. walking around the house) or 8 minutes more moderate activity (4.5 METS e.g. leisurely bicycling) per week. As absolute levels of physical activity are generally low in older adults, a small absolute increase in activity could represent an important relative increase in activity. Furthermore, the dose-response relationship between physical activity and health means even a small increase in physical activity can have a substantial impact on health, particularly in those with low baseline levels of activity (26). Given the aging population in the UK, and the forthcoming retirement of the ‘baby boom’ generation, the aggregate health impacts of increasing physical activity with retirement are likely to be substantial.

There are a number of plausible pathways through which retirement could be associated with an increase in physical activity. With the cessation of formal work, retirees frequently have more free time which could be invested in physically active pursuits including exercise, gardening, volunteering or childcare. Retirement may also be associated with an increase in household physical activities, such as cleaning or chores, which, may offer substantial health benefits (25,27). Retirement could also be associated with a shift to more active forms of transportation, as time constraints associated with travel may be less severe. In England, adults become eligible for a free bus pass when they reach state pension age, which may incentivize greater public transport use in older adults (28–30). Public transport use has been found to be an effective way to incorporate physical activity into daily life and thus movements away from private, passive forms of transportation over retirement could represent a key pathway through which physical activity could increase with retirement (31).

The association between retirement and physical activity differed in magnitude and direction by the activity level of a participant’s past occupation. These differences persisted even after adjusting for wealth and education, thus suggesting that it is the activity level rather than socioeconomic factors associated with the occupation which drive these differences. The present study was unable to distinguish between domains of PA, such as leisure and travel as participants did not provide this information. However, previous reviews have found that the decrease in physical activity in participants retiring from heavy manual labour may be due to a decline in occupational activity that is incompletely compensated for by increases in other activity domains (13).

Participants retiring from sedentary occupations reported the highest magnitude of increase in physical activity. This may be of great public health significance given the transition towards more sedentary, office-based occupations in many service-based economies (32). This study highlights the potential importance of public health interventions targeted at the retirement period, but the existing volume of evidence for such interventions is modest, particularly for workplace-based interventions (33). Interventions to encourage active habits or to increase leisure-time activity in workers engaged in heavy manual labour prior to retirement could help compensate for the decline in occupational activity associated with retirement in this group. Interventions to minimise work-related injury, such as training in lifting techniques, worker exercise programmes to improve strength and flexibility and job redesign could also be effective in improving the capacity of retirees to participate in physical activity in later life (34).

### Study Strengths and Limitations

The ELSA cohort is broadly representative of the English population, except for limited ethnic diversity (35). Between-wave comparisons have highlighted that participants lost to follow-up are on average older, less affluent, less educated, more likely to be from a non-managerial occupation and more likely to suffer from a chronic illness than those with complete follow-up (35). However, unrepresentative participant drop-out is commonly reported in nationwide surveys (36).

Residential environmental factors could have moderated or mediated the association observed in this study. However, in a study of intra-individual changes in physical activity we would have expected any such impacts to be concentrated among participants who moved home over the retirement transition, and no information about residential location or moving home was available in this dataset (37).

Using self-reported retirement status allows for subjectivity in how retirement is defined, which is valuable given the increasingly individualised nature of retirement as a late-life transition. However, ceasing work, drawing a pension and reaching state pension age are not necessarily coincident and thus self-reported retirement status comes at the detriment to inter-individual comparability. This makes it difficult to infer about the forces driving the associations observed, as there may be few definitive elements characterising retirement.

This study assumed that each bout of physical activity was 30 minutes long based on evidence indicating that older adults often accumulate activity in relatively short bouts (38–40). In reality one might expect that ‘mild’ activities may be sustained for longer durations than vigorous activities, thus introducing error that is differential upon activity intensity. However, there is no reason to believe that the duration of physical activity would differ systematically by retirement status or occupational activity level and thus, whilst the assumption of constant activity duration may reduce the accuracy of the magnitude of change in METhrs/wk, it is unlikely to lead to differential bias in the evaluation of the physical activity implications of retirement. The agreement between the categorical and continuous measures of physical activity further supports the conclusion of a relative increase in physical activity with retirement.

Physical activity was self-reported and thus outcome ascertainment is likely to be affected by recall and social desirability bias (41). It is plausible that participants may recall physical activity more accurately after retirement, as with more free-time, physical activity may form a more important and memorable, part of one’s day. This could lead to an over-estimation of the association between retirement and physical activity.

## Conclusion

This study found retirement to be associated with a significant increase in total physical activity. Retirement from an occupation involving heavy manual labour is associated with a significant decrease in physical activity, whereas retirement from a sedentary or standing occupation was found to be associated with a significant increase in activity.

The findings of this study highlight the potential importance of public health interventions targeted at this transitional period, to induce sustainable change in physical activity behaviours (42).

## Supplementary Material

Supplementary Materials

## Figures and Tables

**Table 1:** Baseline sample characteristics stratified by employment/retirement status.

	Total n=10,693	Remained employed n=2,723	Always retired n=5,771	Retired over period n=2,199	P-value
**METhrs/wk**	5.97 (3.62)	6.99 (3.49)	5.19 (3.57)	6.78 (3.43)	< 0.001
**Age**	64.74 (9.80)	54.98 (4.28)	71.10 (8.11)	60.14 (5.16)	< 0.001
**Male Sex**	4,804 (45%)	1,322 (49%)	2,374 (41%)	1,108 (50%)	< 0.001
**White Ethnicity**	10,330 (97%)	2,557 (94%)	5,638 (98%)	2,135 (97%)	< 0.001
**Married/Civil Partnership**	6,961 (65%)	1,834 (67%)	3,506 (61%)	1,621 (74%)	< 0.001
**Highest Education**					< 0.001
No Qualification	2,670 (25%)	340 (13%)	2,006 (35%)	324 (15%)	
Secondary	3,452 (33%)	1,095 (42%)	1,628 (28%)	729 (33%)	
Further	1,494 (14%)	345 (13%)	763 (13%)	386 (18%)	
Degree or equivalent	1,920 (18%)	613 (23%)	768 (13%)	539 (25%)	
Foreign/Other	1,011 (10%)	218 (8%)	583 (10%)	210 (10%)	
**Wealth Quintile**					< 0.001
1 (lowest)	1,744 (16%)	446 (16%)	1,091 (19%)	207 (9%)	
2	2,202 (21%)	685 (25%)	1,112 (19%)	405 (18%)	
3	2,151 (20%)	526 (19%)	1,181 (20%)	444 (20%)	
4	2,284 (21%)	547 (20%)	1,198 (21%)	539 (25%)	
5 (highest)	2,312 (22%)	519 (19%)	1,189 (21%)	604 (27%)	
**Self-reported health**					< 0.001
Poor	606 (6%)	54 (2%)	497 (9%)	55 (3%)	
Fair	1,868 (17%)	304 (11%)	1,328 (23%)	236 (11%)	
Good	3,489 (33%)	850 (31%)	1,941 (34%)	698 (32%)	
Very good	3,258 (30%)	954 (35%)	1,485 (26%)	819 (37%)	
Excellent	1,472 (14%)	561 (21%)	520 (9%)	391 (18%)	
**Mobility: difficulty walking 100 yards**	973 (9%)	38 (1%)	880 (15%)	55 (3%)	< 0.001
**Current smoker**	1,401 (13%)	440 (16%)	677 (12%)	284 (13%)	< 0.001
**Alcohol consumed in last 12 months**	9,458 (88%)	2,511 (92%)	4,888 (85%)	2,059 (94%)	< 0.001

Data are presented as mean (SD) for continuous measures and n (%) for categorical measures. P-values are for ANOVA for continuous measures and Pearson’s chi-squared test for categorical measures.

**Table 2:** Comparison of participants on their last wave before and first wave after retirement.

	Last Wave Before Retirement n=2,001	First Wave After Retirement n=2,001	P-value
**Male Sex**	1,000 (50%)	1,000 (50%)	n/a
**White Ethnicity**	1,949 (97%)	1,949 (97%)	n/a
**Highest Education**			n/a
No Qualification	284 (14%)	284 (14%)	
Secondary	659 (33%)	652 (33%)	
Further	330 (16%)	328 (16%)	
Degree or equivalent	471 (24%)	482 (24%)	
Foreign/Other	257 (13%)	255 (13%)	
**METhrs/wk**	6.72 (3.42)	6.96 (3.47)	0.022
**Age**	63.08 (5.22)	64.71 (4.64)	<0.001
**Married/Civil Partnership**	1,471 (74%)	1,464 (73%)	0.800
**Wealth Quintile**			0.070
1 (lowest)	167 (8%)	180 (9%)	
2	346 (17%)	301 (15%)	
3	422 (21%)	378 (19%)	
4	511 (26%)	546 (27%)	
5 (highest)	555 (28%)	596 (30%)	
**Self-reported health**			0.600
Poor	61 (3%)	64 (3%)	
Fair	230 (11%)	262 (13%)	
Good	672 (34%)	656 (33%)	
Very good	704 (35%)	701 (35%)	
Excellent	334 (17%)	318 (16%)	
**Mobility: difficulty walking 100 yards**	63 (3%)	93 (5%)	0.014
**Current smoker**	215 (11%)	193 (10%)	0.250
**Alcohol consumed in last 12 months**	1,863 (93%)	1,843 (92%)	0.230

Data are presented as mean (SD) for continuous measures, and n (%) for categorical measures. P-values are for two sample t-tests for continuous measures and Pearson’s chi-squared test for categorical measures. Where ‘n/a’ is stated, the samples were identical.

**Table 3:** Linear fixed effect regression with group level adjustment for the association between retirement and physical activity (METhrs/wk) (n = 10,693).

	Model 1:	Model 2:	Model 3:	Model 4:	Model 5:	
	Univariate	Model 1 + Age	Model 2 + Wealth	Model 3 + Health variables (SRH and Mobility)	Model 4 + Behavioural variables (Smoking & Alcohol consumption)	
METhrs/wk	Unadjusted β (95% CI)	β (95% CI)	β (95% CI)	β (95% CI)	β (95% CI)	P-value
**Retirement**	0.036 (-0.069, 0.142)	0.657 (0.545, 0.770)	0.641 (0.528, 0.754)	0.606 (0.494, 0.717)	0.602 (0.490, 0.713)	<0.001
**Age**		-0.122 (-0.131, -0.113)	-0.121 (-0.129, -0.112)	-0.099 (-0.108, -0.090)	-0.097 (-0.106, -0.088)	<0.001
**Wealth**			0.132 (0.078, 0.187)	0.119 (0.065, 0.173)	0.119 (0.065, 0.173)	<0.001
**Self-reported health**				0.371 (0.328, 0.413)	0.369 (0.327, 0.412)	<0.001
**Mobility**				-1.069 (-1.204, -0.933)	-1.060 (-1.196, -0.924)	<0.001
**Smoking**					-0.013 (-0.226, 0.200)	0.905
**Alcohol Consumption**					0.300 (0.147, 0.453)	<0.001
**_cons**	5.925 (5.849, 6.001)	13.837 (13.273, 14.401)	13.332 (12.731, 13.933)	10.803 (10.163, 11.442)	10.401 (9.724, 11.078)	

Data are given for the Beta coefficient associated with retirement with 95% confidence interval. P-values from a Wald test are given for the maximally adjusted model. All models include the same sample of participants. _cons is the constant term in the regression model that equates to the METhrs/wk when the covariates are equal to zero. Stepwise adjustment of single variables is shown in Table S6.

**Table 4:** Baseline comparison of participants by most recent occupational activity level.

	Total	Sedentary Occupation	Standing Occupation	Physical Work	Heavy Manual Labour	
	n=5,109	n=1,837	n=1,418	n=1,477	n=377	P-value
**METhrs/wk**	6.91 (3.45)	6.95 (3.47)	6.85 (3.35)	6.68 (3.44)	7.85 (3.62)	<0.001
**Age in years**	57.67 (5.69)	57.49 (5.78)	57.95 (5.67)	57.85 (5.78)	56.80 (4.80)	0.001
**Male Sex**	2,511 (49%)	848 (46%)	530 (37%)	814 (55%)	319 (85%)	<0.001
**White Ethnicity**	4,874 (95%)	1,750 (95%)	1,339 (94%)	1,416 (96%)	369 (98%)	0.030
**Married/Civil Partnership**	3,588 (70%)	1,317 (72%)	998 (70%)	1,005 (68%)	268 (71%)	0.140
**Highest Education**						<0.001
No Qualification	677 (14%)	114(6%)	183 (13%)	289 (20%)	91 (25%)	
Secondary	1,889 (38%)	671 (38%)	470 (34%)	588 (41%)	160 (43%)	
Further	757 (15%)	217 (12%)	217 (16%)	262 (18%)	61 (17%)	
Degree or equivalent	1,214 (24%)	632 (36%)	380 (27%)	178 (12%)	24 (7%)	
Foreign/Other	449 (9%)	144 (8%)	140 (10%)	133 (9%)	32 (9%)	
**Wealth Quintile**						<0.001
1 (lowest)	667 (13%)	138 (8%)	170 (12%)	287 (19%)	72 (19%)	
2	1,107 (22%)	308 (17%)	313 (22%)	378 (26%)	108 (29%)	
3	1,009 (20%)	344 (19%)	290 (20%)	289 (20%)	86 (23%)	
4	1,132 (22%)	467 (25%)	332 (23%)	283 (19%)	50 (13%)	
5 (highest)	1,194 (23%)	580 (32%)	313 (22%)	240 (16%)	61 (16%)	
**Self-reported health**						<0.001
Poor	104 (2%)	37 (2%)	29 (2%)	31 (2%)	7 (2%)	
Fair	560 (11%)	180 (10%)	141 (10%)	191 (13%)	48 (13%)	
Good	1,604 (31%)	535 (29%)	431 (30%)	502 (34%)	136 (36%)	
Very good	1,855 (36%)	676 (37%)	537 (38%)	524 (35%)	118 (31%)	
Excellent	986 (19%)	409 (22%)	280 (20%)	229 (16%)	68 (18%)	
**Mobility: difficulty walking 100 yards**	95 (2%)	35 (2%)	28 (2%)	29 (2%)	3 (1%)	0.470
**Current smoker**	742 (15%)	198 (11%)	163 (11%)	294 (20%)	87 (23%)	<0.001
**Alcohol consumed in last 12 months**	4,750 (93%)	1,741 (95%)	1,300 (92%)	1,371 (93%)	338 (90%)	<0.001

Data are presented as mean (SD) for continuous measures, and n (%) for categorical measures. P-values are for ANOVA for continuous measures and Pearson’s chi-squared test for categorical measures.

**Table 5:** Fixed effect linear regression stratified by most recent level of occupational activity (n = 5,109).

METhrs/wk	Unadjusted β (95% CI)	P-value	Adjusted β (95% CI)	P-value
**Sedentary Occupation** (n = 1,837)	0.275 (0.105, 0.444)	0.002	0.580 (0.375, 0.785)	<0.001
**Standing Occupation** (n = 1,418)	0.134 (-0.061, 0.329)	0.177	0.330 (0.095, 0.565)	0.006
**Physical Work** (n = 1,477)	-0.230 (-0.441, -0.019)	0.033	0.115 (-0.131, 0.362)	0.359
**Heavy Manual Labour** (n = 377)	-0.803 (-1.346, -0.260)	0.004	-0.823 (-1.440, -0.206)	0.009

Data is given for the Beta coefficient associated with retirement, 95% confidence interval and P-value for a for a Wald test

* Adjusted for participant age, wealth quintile, self-reported health, self-reported mobility, current smoking status and alcohol consumption in the last 12 months
