# Supplementary material for: The physical activity implications of retirement across occupational activity groups
Source: Prev Med. Author manuscript; Available in PMC 2023 Jul 3. (PMC7614714; doi:10.1016/j.ypmed.2023.107570)
Supplement: Supplementary Materials [file EMS177418-supplement-Supplementary_Materials.pdf]

Equation S1. Equation for calculation of METhrs/wk

$$METhrs/wk = \frac{(9 * frequency\ vigorous\ PA) + (4.5 * frequency\ moderate\ PA) + (2 * \ frequency\ mild\ PA)}{2}$$

METhrs/wk, metabolic equivalent of task hours per week; PA, physical activity

Table S1. ELSA physical activities listed under vigorous, moderate and mild headings

**Vigorous**	**Moderate**	**Mild**
For example:	*For example:*	*For example:*
Running or jogging	Gardening	Vacuuming
Swimming	Cleaning the car	Laundry
Cycling	Walking at a moderate pace	Home repairs
Aerobics or gym workout	Dancing	
Tennis	Floor or stretching exercises	
Digging with a spade or shovel		

Physical activity was assessed via three questions in the ELSA questionnaire which asked '*We would like to know the type and amount of physical activity involved in your daily life. Do you take part in sports or activities that are vigorous/moderately energetic/mildly energetic?*' ELSA, English Longitudinal Study of Ageing

Table S2. Frequency of activity per week

ELSA Questionnaire Frequency Categories	Coded Frequency per week
More than once a week	1.5
Once a week	1.0
One to three times a month	0.5
Never or hardly ever	0.0

ELSA, English Longitudinal Study of Ageing

Table S3. Categorisation of activity into high, moderate, low and inactive categories

Level of physical activity	Criteria
High	Vigorous activity more than once per week
Moderate	Vigorous activity once a week or vigorous activity less than once a week and moderate activity at least once a week
Low	Vigorous activity less than once a week, moderate activity less than once a week, and mild activity at least once a week
Inactive	No vigorous, moderate or mild activity on a weekly basis

Table S4. Percentage and patterns of missingness in full ELSA sample (waves 4–9) (52,237 observations)

	Percentage Missingness in ELSA Sample (%)	Patterns of Missingness
Wealth Quintile	15.11	7,894 not imputed, non-sample member or institutional respondent
Age	0.00	n/A
Physical activity	0.03	5 refused<br>9 don't know<br>1 no self-completion interview
Sex	0.00	n/A
Self-reported health	5.07	6 refused<br>13 don't know<br>2,630 not applicable
Mobility	0.03	9 refused<br>9 don't know
Current Smoker	0.87	1 refused<br>30 don't know<br>65 capi/Interview error<br>356 not applicable
Alcohol consumption	15.05	705 not answered<br>77 no self-completion interview<br>4,650 schedule not applicable<br>2,428 not applicable
Education	1.99	178 refused<br>214 don't know<br>647 not asked
Ethnicity	0.06	19 refused<br>7 don't know<br>5 schedule not applicable
Marital Status	0.00	n/A

Missingness as defined in ELSA data dictionaries. Capi, computer-assisted interviewing; ELSA, English Longitudinal Study of Aging; n/A, Not applicable.

Table S5. Comparison of observations with and without missing data for the covariates included in the multivariable regression model

	Total Obs. = 52,237 (n = 14,067)	Missing Obs. =13,805 (n = 3,374)	Complete Obs. = 38,432 (n = 10,693)	P-value
METhrs/wk	5.86 (3.68)	5.62 (3.89)	5.95 (3.59)	
Age	67.45 (10.17)	65.01 (12.05)	68.33 (9.25)	< 0.001
Male Sex	24,234 (46%)	6,617 (48%)	17,617 (46%)	0.015
White Ethnicity	50,416 (97%)	12,952 (94%)	37,464 (98%)	<0.001
Married/Civil Partnership	34,621 (66%)	9,576 (69%)	25,045 (65%)	<0.001
Highest Education				<0.001
No Qualification	11,927 (23%)	3,423 (26%)	8,504 (22%)	
Secondary	16,475 (32%)	4,232 (32%)	12,243 (32%)	
Further	7,367 (14%)	1,612 (12%)	5,755 (15%)	
Degree or equivalent	9,803 (19%)	2,502 (19%)	7,301 (19%)	
Foreign/Other	5,638 (11%)	1,358 (10%)	4,280 (11%)	
Wealth Quintiles				<0.001
1 (lowest)	6,719 (15%)	1,458 (25%)	5,261 (14%)	
2	8,534 (19%)	1,305 (22%)	7,229 (19%)	
3	9,389 (21%)	1,172 (20%)	8,217 (21%)	
4	9,747 (22%)	973 (17%)	8,774 (23%)	
5 (highest)	9,927 (22%)	976 (17%)	8,951 (23%)	
Self-reported Health				<0.001
Poor	2,933 (6%)	824 (7%)	2,109 (5%)	
Fair	8,830 (18%)	2,017 (18%)	6,813 (18%)	
Good	16,409 (33%)	3,479 (31%)	12,930 (34%)	
Very good	15,090 (30%)	3,309 (30%)	11,781 (31%)	
Excellent	6,340 (13%)	1,541 (14%)	4,799 (12%)	
Mobility: difficulty walking 100 yards	5,718 (11%)	1,895 (14%)	3,823 (10%)	<0.001
Current smoker	5,537 (11%)	1,755 (13%)	3,782 (10%)	<0.001
Alcohol consumed in last 12 months	39,107 (88%)	5,425 (91%)	33,682 (88%)	<0.001

Data are presented as mean (SD) for continuous measures, and n (%) for categorical measures. Obs. refers to the entry for a single wave. n refers to the number of participants. Each participant may contribute more than one observation. P-values are gained from a logistic regression model, regressing missingness against each covariate separately, adjusting for clustering by participant. METhrs/wk, metabolic equivalent of task hours per week; Obs., observations; SD, standard deviation

Table S6. Linear fixed effect regression with single variable adjustment for the association between retirement and physical activity (METhrs/wk)

	Univariate	Univariate + Age	Univariate + Wealth	Univariate + Self-reported health	Univariate + Mobility	Univariate + Smoking	Univariate + Alcohol consumption
METhrs/wk	Unadjusted β (95% CI)	Unadjusted β (95% CI)	Unadjusted β (95% CI)	Unadjusted β (95% CI)	Unadjusted β (95% CI)	Unadjusted β (95% CI)	Unadjusted β (95% CI)
Retirement	0.036 (-0.069, 0.142)	0.657 (0.545, 0.770)	0.023 (-0.082, 0.129)	0.103 (-0.002, 0.207)	0.071 (-0.034, 0.175)	0.047 (-0.059, 0.152)	0.053 (-0.052, 0.158)
Age		-0.122 (-0.131, -0.113)					
Wealth			0.179 (0.124, 0.235)				
Self-reported health				0.497 (0.455, 0.540)			
Mobility					-1.444 (-1.580, -1.308)		
Smoking						0.343 (0.125, 0.560)	
Alcohol							0.656 (0.501, 0.811)

Data is given for the Beta coefficient associated with retirement with 95% confidence interval. All models include the same sample of participants (n = 10, 693). CI, confidence interval; METhrs/wk, metabolic equivalent of task hours per week.

Table S7. Fixed effect multinomial logistic regression demonstrating the relative risk of low, moderate and high physical activity compared to being inactive (n = 5,577)

	Inactive	Low				Moderate				High			
Physical activity level		Unadjusted RR (95% CI)	P-value	Adjusted RR (95% CI)	P-value	Unadjusted RR (95% CI)	P-value	Adjusted RR (95% CI)	P-value	Unadjusted RR (95% CI)	P-value	Adjusted RR (95% CI)	P-value
Retirement	Ref.	1.146 (0.843, 1.558)	0.384	1.473 (1.050, 2.066)	0.025	0.909 (0.689, 1.199)	0.500	1.928 (1.413, 2.631)	<0.001	0.967 (0.719, 1.300)	0.825	2.827 (2.025, 3.947)	<0.001
Age	Ref.			0.988 (0.967, 1.010)	0.287			0.908 (0.889, 0.927)	<0.001			0.860 (0.840, 0.881)	<0.001
Wealth	Ref.			1.066 (0.933, 1.219)	0.347			1.220 (1.077, 1.383)	0.002			1.267 (1.100, 1.460)	0.001
Self-reported health	Ref.			1.108 (1.003, 1.223)	0.043			1.439 (1.309, 1.582)	<0.001			1.848 (1.658, 2.059)	<0.001
Mobility	Ref.			0.509 (0.415, 0.625)	<0.001			0.217 (0.175, 0.270)	<0.001			0.169 (0.117, 0.242)	<0.001
Smoking status	Ref.			1.154 (0.737, 1.806)	0.531			1.277 (0.827, 1.969)	0.270			1.069 (0.630, 1.815)	0.804
Alcohol consumption	Ref.			1.183 (0.901, 1.554)	0.227			1.337 (1.011, 1.767)	0.042			1.843 (1.267, 2.683)	0.001

*The adjusted multivariable model adjusted for participant age, wealth quintile, self-reported health, self-reported mobility, current smoking status and alcohol consumption in the last 12 months. The stated P-values are for a Wald test. CI, confidence interval; Ref, reference (RR=1.0); RR, relative risk

Table S8. Fixed effect multinomial logistic regression demonstrating the relative risk of low, moderate and high physical activity compared to being inactive stratified by past occupational activity level (n = 2,521)

	Inactive	Low				Moderate				High			
Physical activity level	RR (95% CI)	Unadjusted RR (95% CI)	P-value	Adjusted RR (95% CI)	P-value	Unadjusted RR (95% CI)	P-value	Adjusted RR (95% CI)	P-value	Unadjusted RR (95% CI)	P-value	Adjusted RR (95% CI)	P-value
Sedentary Occupation (n = 843)	Ref.	1.309 (0.742, 2.311)	0.353	1.222 (0.589, 2.536)	0.590	1.093 (0.650, 1.838)	0.736	1.422 (0.728, 2.778)	0.302	1.432 (0.822, 2.495)	0.205	2.301 (1.130, 4.687)	0.022
Standing Occupation (n = 688)	Ref.	0.917 (0.512, 1.644)	0.771	1.083 (0.497, 2.358)		0.932 (0.547, 1.587)	0.794	1.306 (0.630, 2.707)	0.472	1.123 (0.638, 1.977)	0.688	1.764 (0.816, 3.814)	0.149
Physical Work (n = 770)	Ref.	1.401 (0.812, 2.416)	0.226	1.110 (0.574, 2.147)	0.756	0.826 (0.509, 1.341)	0.439	0.908 (0.505, 1.631)	0.746	0.731 (0.433, 1.232)	0.239	0.998 (0.530, 1.880)	0.995
Heavy Manual Labour (n= 220)	Ref.	0.646 (0.200, 2.081)	0.464	0.526 (0.122, 2.268)	0.389	0.403 (0.154, 1.058)	0.065	0.301 (0.082, 1.107)	0.071	0.287 (0.104, 0.791)	0.016	0.211 (0.055, 0.809)	0.023

*Adjusted for participant age, wealth quintile, self-reported health, self-reported mobility, current smoking status and alcohol consumption in the last 12 months. The stated P-value is for a Wald test. CI, confidence interval; Ref, reference (RR=1.0); RR, relative risk

Table S9. Changes in the percentage of people meeting physical activity recommendations (11 METhrs/wk).

	Sedentary occupation	Standing occupation	Physical work	Heavy manual labour
Last wave before retirement	852 (46%)	710 (50%)	793 (54%)	215 (57%)
First wave after retirement	985 (54%)	708 (50%)	684 (46%)	162 (43%)

METhrs/wk, metabolic equivalent of task hours per week

Table S10. Multivariable regression including month of questionnaire completion for METhrs/wk (n =10,639)

	Not including month		Including month	
METhrs/wk	Adjusted β (95% CI)	P-value	Adjusted β (95% CI)	P-value
Retired	0.601 (0.489, 0.713)	<0.001	0.601 (0.489, 0.712)	<0.001
Age	-0.068 (-0.106, -0.088)	<0.001	-0.098 (-0.107, -0.089)	<0.001
Wealth	0.119 (0.065, 0.173)	<0.001	0.119 (0.065, 0.173)	<0.001
Self-reported health	0.369 (0.327, 0.412)	<0.001	0.369 (0.326, 0.412)	<0.001
Self-reported mobility	-1.060 (-1.196, -0.924)	<0.001	-1.065 (-1.201, -0.929)	<0.001
Smoking status	-0.013 (-0.226, 0.200)	0.905	-0.014 (-0.227, 0.199)	0.897
Alcohol consumption	0.300 (0.147, 0.453)	<0.001	0.298 (0.145, 0.451)	<0.001
Month				
February			-0.027 (-0.156, 0.102)	0.683
March			0.208 (0.045, 0.372)	0.013
April			0.360 (0.129, 0.591)	0.002
May			0.180 (-0.082, 0.443)	0.178
June			0.001 (-0.162, 0.164)	0.990
July			0.083 (-0.062, 0.227)	0.262
August			0.226 (0.081, 0.371)	0.002
September			0.222 (0.086, 0.359)	0.001
October			0.198 (0.074, 0.322)	0.002
November			0.087 (-0.028, 0.203)	0.139
December			-0.021 (-0.151, 0.109)	0.752
_cons	10.401 (9.724, 11.078)		10.399 (9.707, 11.092)	

*The adjusted multivariable model adjusted for participant age, wealth quintile, self-reported health, self-reported mobility, current smoking status and alcohol consumption in the last 12 months. Data is given for the Beta coefficients associated with retirement, 95% confidence interval and P-value for a Wald test. _cons is the constant term in the regression model that equates to the METhrs/wk when the covariates are equal to zero. CI, confidence interval; METhrs/wk, metabolic equivalent of task hours per week

Table S11. Multivariable regression including wave number for METhrs.wk (n = 10,639)

	Not including Wave		Including Wave	
METhrs/wk	Adjusted β (95% CI)	P-value	Adjusted β (95% CI)	P-value
Retired	0.601 (0.489, 0.713)	<0.001	0.601 (0.489, 0.713)	<0.001
Age	-0.068 (-0.106, -0.088)	<0.001	0.017 (-0.079, 0.113)	0.728
Wealth	0.119 (0.065, 0.173)	<0.001	0.118 (0.064, 0.172)	<0.001
Self-reported health	0.369 (0.327, 0.412)	<0.001	0.370 (0.327, 0.413)	<0.001
Self-reported mobility	-1.060 (-1.196, -0.924)	<0.001	-1.063 (-1.199, -0.928)	<0.001
Smoking status	-0.013 (-0.226, 0.200)	0.905	-0.010 (-0.223, 0.204)	0.928
Alcohol consumption	0.300 (0.147, 0.453)	<0.001	0.300 (0.147, 0.453)	<0.001
Wave				
5			-0.221 (-0.433, -0.008)	0.042
6			-0.369 (-0.757, 0.020)	0.063
7			-0.600 (-1.180, -0.020)	0.043
8			-0.889 (-1.663, -0.116)	0.024
9			-1.165 (-2.139, -0.191)	0.019
_cons	10.401 (9.724, 11.078)		3.132 (-3.001, 9.265)	

*The adjusted multivariable model adjusted for participant age, wealth quintile, self-reported health, self-reported mobility, current smoking status and alcohol consumption in the last 12 months. Data is given for the Beta coefficients associated with retirement, 95% confidence interval and P-value for a Wald test. _cons is the constant term in the regression model that equates to the METhrs/wk when the covariates are equal to zero. CI, confidence interval; METhrs/wk, metabolic equivalent of task hours per week

Table S12. Baseline (first complete wave) comparison of people experiencing unidirectional retirement compared to reversible retirement

	Total (n = 10,693)	Unidirectional (n = 10,385)	Reversible (n = 277)	P-value
METhrs/wk	6.78 (3.43)	6.76 (3.42)	6.92 (3.52)	0.450
Age	60.14 (5.16)	59.84 (4.96)	62.23 (5.94)	<0.001
Male Sex	1,108 (50%)	954 (50%)	154 (56%)	0.064
White Ethnicity	2,135 (97%)	1,864 (97%)	271 (98%)	0.430
Married/Civil Partnership	6,961 (65%)	6,730 (65%)	231 (75%)	<0.001
Highest Level of Education				0.084
No Qualification	324 (15%)	278 (15%)	46 (17%)	
Secondary	729 (33%)	652 (34%)	77 (28%)	
Further	386 (18%)	341 (18%)	45 (16%)	
Degree or equivalent	539 (25%)	455 (24%)	84 (30%)	
Foreign/Other	210 (10%)	185 (10%)	25 (9%)	
Wealth Quintile				0.016
1 (lowest)	207 (9%)	178 (9%)	29 (10%)	
2	405 (18%)	363 (19%)	42 (15%)	
3	444 (20%)	392 (20%)	52 (19%)	
4	539 (25%)	483 (25%)	56 (20%)	
5 (highest)	604 (27%)	506 (26%)	98 (35%)	
Health				0.920
Poor	55 (3%)	50 (3%)	5 (2%)	
Fair	236 (11%)	207 (11%)	29 (10%)	
Good	698 (32%)	606 (32%)	92 (33%)	
Very good	819 (37%)	718 (37%)	101 (36%)	
Excellent	391 (18%)	341 (18%)	50 (18%)	
Mobility: difficulty walking 100 yards	55 (3%)	49 (3%)	6 (2%)	0.700
Current smoker	284 (13%)	248 (13%)	36 (13%)	0.970
Alcohol consumed in last 12 months	2,059 (94%)	1,796 (93%)	263 (95%)	0.340

Data are presented as mean (SD) for continuous measures, and n (%) for categorical measures. P-values are for two-sampled t-test for continuous measures and Pearson's chi-squared test for categorical measures. METhrs/wk, metabolic equivalent of task hours per week; SD, standard deviation

Table S13. Multivariable fixed effect linear regression of the association between retirement and physical activity (METhrs/wk) stratified by whether participants reporting reversible retirement were included

	Including reversible retirement (n =10,639)		Excluding reversible retirement (n =10,276)	
METhrs/wk	β (95% CI)	P-value	β (95% CI)	P-value
Retirement	0.601 (0.489, 0.713)	<0.001	0.642 (0.519, 0.764)	<0.001
Age	-0.068 (-0.106, -0.088)	<0.001	-0.100 (-0.109, -0.091)	<0.001
Wealth	0.119 (0.065, 0.173)	<0.001	0.116 (0.061, 0.171)	<0.001
Self-reported health	0.369 (0.327, 0.412)	<0.001	0.362 (0.319, 0.406)	<0.001
Mobility	-1.060 (-1.196, -0.924)	<0.001	-1.083 (-1.220, -0.945)	<0.001
Smoking status	-0.013 (-0.226, 0.200)	0.905	-0.002 (-0.220, 0.216)	0.987
Alcohol consumption	0.300 (0.147, 0.453)	<0.001	0.297 (0.141, 0.452)	<0.001
_cons	10.401 (9.724, 11.078)		10.588 (9.891, 11.285)	

Data is given for the Beta coefficient associated with retirement, 95% confidence interval and P-value for a Wald test. Models are adjusted for participant age, wealth quintile, self-reported health, self-reported mobility, current smoking status and alcohol consumption in the last 12 months. _cons is the constant term in the regression model that equates to the METhrs/wk when the covariates are equal to zero. CI, confidence interval; METhrs/wk, metabolic equivalent of task hours per week.

Table S14. Comparison of Employed, Semi-Retired and Retired

	Employed Obs. = 18,683 (n = 6,356)	Retired Obs. = 33,266 (n = 9,870)	Semi-Retired Obs. = 303 (n = 273)	P-value
Quintiles of Total wealth	3.23 (1.36)	3.15 (1.38)	3.33 (1.42)	<0.001
Age (years)	58.27 (6.20)	72.63 (8.15)	65.47 (6.10)	<0.001
METhrs/wk	6.96 (3.47)	5.24 (3.65)	6.08 (3.71)	<0.001
Male Sex	9,133 (49%)	14,911 (45%)	198 (65%)	0.002
Self-reported Health				<0.001
Poor	350 (2%)	2,569 (8%)	14 (5%)	
Fair	1,954 (11%)	6,844 (22%)	34 (12%)	
Good	5,604 (32%)	10,716 (34%)	91 (31%)	
Very good	6,346 (36%)	8,646 (27%)	99 (34%)	
Excellent	3,291 (19%)	2,997 (9%)	53 (18%)	
Mobility: difficulty walking 100 yards	368 (2%)	5,336 (16%)	15 (5%)	<0.001
Current smoker	2,567 (14%)	2,946 (9%)	25 (8%)	<0.001
Alcohol consumption in last 12 months	14,424 (93%)	24,440 (86%)	248 (94%)	<0.001
Highest Level of Education				<0.001
None	2,410 (13%)	9,462 (29%)	55 (19%)	
Some	11,400 (63%)	17,926 (55%)	162 (55%)	
Degree or equivalent	4,279 (24%)	5,449 (17%)	80 (27%)	
White Ethnicity	17,687 (95%)	32,451 (98%)	292 (96%)	<0.001
Married/Civil Partnership	13,739 (74%)	20,690 (62%)	204 (67%)	<0.001

Data are presented as mean (SD) for continuous measures, and n (%) for categorical measures. P-values are gained from a logistic regression model, regressing retirement status against each covariate separately and adjusting for clustering by participant. METhrs/wk, metabolic equivalent of task hours per week; Obs., observations; SD, standard deviation

Table S15. Multivariable fixed effect linear regression of the association between retirement and physical activity (METhrs/wk) stratified by whether participants reporting to be semi-retired were included

	Including semi-retired (n = 10,639)		Excluding semi-retired (n = 10,366)	
METhrs/wk	β (95% CI)	P-value	β (95% CI)	P-value
Retirement	0.601 (0.489, 0.713)	<0.001	0.596 (0.482, 0.709)	<0.001
Age	-0.068 (-0.106, -0.088)	<0.001	-0.097 (-0.106, -0.088)	<0.001
Wealth	0.119 (0.065, 0.173)	<0.001	0.126 (0.072, 0.180)	<0.001
Self-reported health	0.369 (0.327, 0.412)	<0.001	0.369 (0.326, 0.412)	<0.001
Mobility	-1.060 (-1.196, -0.924)	<0.001	-1.068 (-1.204, -0.932)	<0.001
Smoking status	-0.013 (-0.226, 0.200)	0.905	-0.009 (-0.223, 0.205)	0.935
Alcohol consumption	0.300 (0.147, 0.453)	<0.001	0.295 (0.141, 0.448)	<0.001
_cons	10.401 (9.724, 11.078)		10.381 (9.702, 11.061)	

Data is given for the Beta coefficient associated with retirement, 95% confidence interval and P-value for a Wald test. Models are adjusted for participant age, wealth quintile, self-reported health, self-reported mobility, current smoking status and alcohol consumption in the last 12 months. _cons is the constant term in the regression model that equates to the METhrs/wk when the covariates are equal to zero. CI, confidence interval; METhrs/wk, metabolic equivalent of task hours per week.
